# Influence of Dental Malocclusion on Body Posture and Foot Posture in Children: A Cross-Sectional Study

**DOI:** 10.3390/healthcare8040485

**Published:** 2020-11-14

**Authors:** Ana Juana Pérez-Belloso, Manuel Coheña-Jiménez, Maria Eugenia Cabrera-Domínguez, Antonio Francisco Galan-González, Antonia Domínguez-Reyes, Manuel Pabón-Carrasco

**Affiliations:** 1 Department of Podiatry, Faculty of Nursing, Physiotherapy and Podiatry, University of Sevilla, 41009 Sevilla, Spain; aperez30@us.es (A.J.P.-B.); mpabon2@us.es (M.P.-C.); 2 Department of Podiatry, University of Sevilla, 41009 Sevilla, Spain; 3 Faculty of Dentistry, University of Sevilla, 41009 Sevilla, Spain; mcabrera@us.es (M.E.C.-D.); agalan@us.es (A.F.G.-G.); adominre@us.es (A.D.-R.)

**Keywords:** children, dental malocclusions, foot postures, temporomandibular joint disorders, pressure

## Abstract

The number of studies that investigate the correlations between the temporomandibular system and body posture, postural control, or the distribution of plantar pressure has recently been increasing. However, most of the existing information is not conclusive. Therefore, the study objective was to evaluate if the features of dental malocclusion are correlated with body posture alterations at the lower limb level. This is a multicentre cross-sectional study with 289 children (8–14 years). Angle’s molar relation was analysed at the dental level. The postural control and the plantar pressure distribution were recorded via a force platform. Correlation and inferential analysis between the Angle class and the foot’s biomechanics were tested. The centre of gravity is anteriorised in Angle’s Class II in both the molar class (*p* ≤ 0.001) and the canine class (*p* ≤ 0.001). Likewise, a relationship was observed between the contact surface and Angle’s classes, being higher in class III than in II (*p* ≤ 0.001). The plantigrade phase is shortened in Angle’s Class III. A relationship was found between Angle’s Class II and a forward movement of the centre of gravity. No relationship was found between the Foot Posture Index and the truncated scaphoid height and the dental classification. An evident relationship between the gait typology and dental malocclusion was not found.

## 1. Introduction

Many researchers have investigated the various factors that can influence body posture: mood states, anxiety, head and neck positions, the tongue, oral functions (respiration, swallowing), oculomotor and visual systems, and the inner ear. Similarly, attempts have been made to determine the relationship of the stomatognetic system, body posture, and the distribution of plantar pressure [1,2,3,4,5,6]. However, most of the existing information is not conclusive, there being a specific uncertainty with results for and against [7,8,9,10,11,12,13,14] these correlations and without evident clinical relevance [15].

The human posture is the result of the body’s position and the spatial relations between its anatomical segments, in equilibrium with movement and gravity [16]. Postural adjustments are added to this. These are small changes that take place in the posture caused by the entry of visual, vestibular, and somatosensorial stimuli integrated in a complex regulating system [11]. Studies demonstrate that breathing, the position of the head, the neck, moods, and anxiety can modify posture [16,17,18,19]. Those authors who mean to demonstrate the correlation between the distinct body segments base themselves on the hypothesis that any functional alteration of the oral cavity, which involves an alteration of the biomechanics of the temporomandibular articulation (TMJ), produces an alteration of the functions of masticatory muscles, which can be transmitted to all the distal muscles through muscle chains [20,21]. This transmission through the muscle chains brings about alterations of the cervical muscles, producing changes in all the planes of the spinal column’s space at this level, provoking descending compensations at both the dorsal and the lumbar level. As a consequence, sacral deviations could cause, if the articular spaces do not cede enough, pelvic deviations and rotations with the resulting compensations at the level of the hip and the lower limb [22].

Most of the studies found are oriented to demonstrating the effects that dental occlusion has on the head and body, and there are very few data published about how postural alterations are related with alterations in TMJ biomechanics and, therefore, alterations at the level of dental occlusion. It is easier to compensate a postural alteration when dental occlusion problems do not exist [10]. Other authors reveal that the force vectors of these muscle chains not only act in a descending direction. Through the kinetic chains that make up our biomechanical system, any alteration at the lower limb level could bring about ascending compensations and even provoke alterations in the TMJ [23,24] and, through this, dysfunctions in the way of inter-relating the upper arch and lower arches (what is known as dental occlusion) and changes at the postural level.

This relationship is associated with various studies that show that pronation of the foot causes an internal rotation of the tibia, which is accompanied by an internal rotation of the femur, an anteriorization of the pelvis, and the consequent structural changes in the spine. This is termed by some authors as the global pronation of the complete global limb [23,24] and, therefore, dysfunctions in the way the upper arches and lower arches interrelate (which is known as dental occlusion) and consequently alter craniofacial growth and its development are of great importance in the establishment or severity of dentomaxillary abnormalities [21].

Finally, there are few studies that have tried to correlate dental malocclusion with postural disorders and most of them have been hampered by a series of limitations, such as the paucity of subjects or very unequal groups, incomplete sample descriptions, and a limited number of parameters and/or conditions tested [8,9,13,15]. Due to the clinical impact that a correlation between dental malocclusion and the body posture can have, and the scant information available concerning the topic, additional research is justified. This study assessed if malocclusion features are correlated with body posture at a detectable level.

Therefore, the purpose of this study was to evaluate the presence of a possible association between plantar pressure, stabilometry, and dental malocclusion in children aged 8 to 14 years.

## 2. Materials and Methods

### 2.1. Study Design and Subjects

A cross-sectional analysis study was done. The total sample was of 289 children (158 boys and 131 girls) aged from 8–14 years old. When selecting the subjects, it was taken into account that malocclusions begin at an early age, being present in approximately 50% of primary teeth, in some cases reaching 70% [25]. As soon as the primary teeth are completed, some alteration of antero-posterior malocclusion may be detected and early and multidisciplinary intervention in the children carried out, avoiding more traumatic and costly treatments in adult ages [26]. These children were recruited during 2019 from a public sector school randomly selected in the province of Seville, Spain by a sequence generator [27]. The Strengthening the Reporting of Observational Studies in Epidemiology verification list was used.

The participants have to fulfil the following inclusion criteria: children aged from 8 to 14 years old and informed consent provided by the parents or legal tutor. The parents/tutors were previously informed concerning the study. The exclusion criteria were previous surgery of the lower limbs or the upper part of the body; having orthodontic or orthopaedic treatment, both fixed and removable; previous severe trauma that altered the child’s initial posture; and lacking enough teeth to determine the dental classification.

### 2.2. Assessments and Procedure

For greater precision, 3 variables related with the foot were valued: foot posture, to detect alterations in the 3 planes; the height of the truncated scaphoid; and plantar pressures. Data concerning the gravity centre variable were added to all this.

The biomechanical exploration was done in all the cases by the main researcher using the Foot Posture Index (FPI) as a method validated and recommended by diverse authors. This is considered a supplementary clinical tool for the valuation of podiatry alterations. It consists of quantifying the degree of a foot’s neutral, pronated, or supinated position [28,29]. This tool has 6 points for the clinical evaluation, measuring the multisegmental nature of the foot’s posture in 3 planes, and does not require the use of specialised equipment [30]. The participants were evaluated in a relaxed position, stood on a bench at a height of 40 cm to facilitate the measurement [28,31]. The FPI was measured by an expert podiatrist who did not know the results obtained by the dentist.

The height of the truncated scaphoids is calculated dividing the scaphoid height by the truncated length of the footprint; that is to say, the length of the plantar print except the toes, which coincides with the metatarsophalangeal joint. Distinct studies found that this measurement of the scaphoid is that which has presented a greater correlation with the angular measurements done by X-ray [32].

Another series of data was obtained via baropodometric analysis. This enables finding out the distribution of the loads or pressures in different plantar zones and evaluating the direct influences of the forces applied in the three periods of the support phase, as well as their intensity and duration. It allows valuating the areas of greater pressure and reflects the changes, which are produced in the static and dynamic support, as well as the pressure peaks, which are formed in the support phase by the time unit. The baropodometric measurements are done with a T-plate^®^ pressureplatform (Medicapteurs, Balma, France). This is a platform with a useful area of 40 × 40 cm and a flat surface only 8 mm thick. The measurement instrument was placed, but not fixed, on a hard and rigid surface. It has resistive-type sensors, which do not require calibration. The data are sent digitally to a computer and are then processed by a software, which shows the plantar pressure parameters (kPa), contact time (s) and pace (steps/minute). The device recorded the distribution of the plantar pressure on the first and fifth metatarsal heads and the calcaneus, both in the dynamic and in the static situation. The measurement was done twice for a greater precision of the data [33].

For the static analysis, the subjects remained barefoot on the sensor’s sphere in a comfortable posture, with their arms relaxed at the side of the trunk. The subjects were asked to keep as still as possible and to maintain a relaxed posture of the head and the body. During the measurement period, they were asked to look at a mark on the wall at the height of their eyes. This mark had a diameter of 1 cm. The distance between the subjects and the wall was 2 metres. The subjects were not allowed to move their feet during the test.

The material used to carry out the dental explorations was what is usual in an epidemiological study of oral health: intraoral mirrors, dental probes, antiseptic solution, recipients for material, gloves, mask, paper towels and calibrators (millimetre caliper and ruler). Angle’s molar relation was analysed, considering it as Class I when the mesiovestibular cuspid of the first upper molar superior occluded in the vestibular groove of the first lower molar; as Class II when the mesiovestibular cuspid of the first upper molar superior occluded in front of the vestibular groove of the first lower molar; and as Class III when this cuspid occluded behind the vestibular groove of the lower molar [34]. The dental examinations were done in natural light, in accordance with the recommendations of the WHO. Molar classes I, II, and III were recorded using the first permanent molars as reference teeth. The cases of displacement of half the cuspid less than the normal was considered Class I [35].

With the aim of not troubling or interfering in the school timetable, the days and times for the exploration were set in advance, establishing small groups of students who, methodically and under the control of a tutor of the centre, went into the room that was used for the exploration. To avoid interoperator bias, all the dental examinations were done by a single dental specialist. Before beginning the study, a questionnaire was given to the parents/tutors of the children. The questionnaire asked about aspects such as the presence of previous traumas, prior dental treatment, and the use of plantar supports. After this first filter, an examination was done by dental specialists and podiatrists, where those children who did not fulfil the inclusion criteria were excluded.

### 2.3. Ethical Considerations

This study was approved by the Biomedical Research Ethics Committee (code number: 0484-M1-19) of the Junta de Andalucía (Andalusian Regional Government). The ethical standards for human research stipulated in the Declaration of Helsinki (World Medical Association), in the Council of Europe Convention on Human Rights and Biomedicine, in the UNESCO Universal Declaration on the Human Genome and Human Rights, and in similar institutional declarations were observed at all times. Participation was voluntary. No incentives were offered for participation in the study.

### 2.4. Statistical Analysis

The study was designed to detect changes that exceeded 0.8 (high effect size) for a variation of the sample in accordance with the previous classification, with a type I error of 0.05 and a type II error of 0.2. This calculation meant a necessary sample size of 232 subjects. A total of 289 subjects were recruited.

Data exploration was done for the statistical analysis, generating summary statistics for all the cases. This procedure is used to identify atypical or extreme values and characterise differences between case groups. It likewise enables identifying if the statistical techniques considered are appropriate and indicates the need to transform the data or use non-parametric tests. The numerical variables (quantitative) are summarised with averages and standard deviations, while the non-numerical variables (qualitative) have frequencies and percentages. Statistical analysis was performed by SPSS 25 (IBM Corp., Armonk, NY, USA). The Kolmogorov–Smirnov test was applied to determine the distribution of the variables. The chi-squared test was used in the case of the qualitative variables. The ANOVA parametric test was applied to the FPI variables; the truncated scaphoid height, plantar pressures, and the angle classification whenever conditions of normality were met. When this was not so, the Kruskal–Wallis test was employed. The bivariate relation between plantar pressures and FPI was determined via the Pearson correlation test or the Spearman test when there was not normality. The strength of association between the qualitative variables was done via Cramer’s V. The level of significance adopted for all the statistical analyses was *p* < 0.05.

## 3. Results

### Sample Characteristics

The study sample was composed of 289 participants (158 boys and 131 girls). The average FPI for the left foot was 4.83 ± 2.28, that of the height of the truncated scaphoid was 2.58 ± 0.72. For the right foot, the FPI was 5.04 ± 2.27 and the height of the truncated scaphoid was 2.57 ± 0.76. The foot measurements were obtained at both a barometric level (static and dynamic) and a positioning level (FPI and scaphoid height). The barometric data of both feet and the degree of association between the FPI, the scaphoid height, and the static and dynamic pressures are presented (Table 1).

As to the oral cavity, 67.5% have nasal breathing, 30.1% have mixed breathing, and only 2.4% mouth breathing. The presence of macroglossia is valued when there is a relation with Angle’s Class III. This was seen in 2.42% of the participants. It was also revealed that 13.1% have tongue thrusting when swallowing. A descriptive analysis of the stomatological data was done. Table 2 shows the degree of relation of the measurements taken and their strength of association is valued as well. A predominance of Angle’s Class II was seen in both its molar and canine versions. There is a strong association when comparing within the same class and an average one when comparing the canine class with the molar class. On the other hand, no relationship was found between the hip position and the Angle classes (*p* value ≥ 0.05).

Table 3 shows the inferential analysis. A valuation was done in both static and dynamic situations to infer possible relations between the stomatological apparatus and the foot’s biomechanics.

In our analysis of the foot variables in comparison with the dental classification, no relationship was found between the FPI and the truncated scaphoid height and the dental classification with *p* ≥ 0.05. A relationship only exists in the left foot’s FPI and the right foot’s scaphoid height. Regarding the barometric analysis compared to the dental classification, there were significant findings with the contact surface, the plantigrade phase and especially the centre of gravity and the Angle class (Table 4). A predominance is seen of the anteriority of gravity in Angel’s Class II. In contrast, the Class I of centre of gravity was posteriorly located, the same as with Class III. The intensity of association has a moderate level.

## 4. Discussion

In this study, it was found that the highest percentage of the children studied have their subtalar joint in a valgus position, irrespective of their being cavus or not. A percentage of 38.4 of the sample were valgus, 45.0% cavus–valgus, and 16.6% were normal. This is directly related with the genus typologies. Here, it is seen that 80.3% have knee valgus. The clinical evidence demonstrates that the valgus position of the subtalar joint produces an internal rotation of the tibia, and thereby a change in the direction of the force vectors of the musculature which is in it. This begins to produce an ascending biomechanical disorder both at the skeletal structural level and of the muscular and fascia system. Arazi et al. define normal physiological genus valgus as happening during childhood (2–6 years old). Above this age range and having abnormal values, it is not then considered physiological. It clears up as the person grows [36]. In our study, the participants were between 8–14 years old, so those who have genus valgus could not be considered as having physiological genus valgus.

According to Khamis and Yizhar, this means that the knee’s joint has a tendency to deviate in valgus, helped largely by the ground’s forces of reaction and the deviation of the axes of the joints’ normal loads [37]. Taking into account that there is a multitude of biomechanically associated anatomical structures in the foot’s movement towards the TMJ, data relative to the genus and the pelvis were taken, as the compensations could take place in any of the articular structures, which are in the lower limb and trunk. The pelvic anteversion produces an alteration of the musculature intimately related at the cervical level and changes in the direction of the centre of gravity are possible, reflected in the pressure platform. Interestingly, our study revealed that, in relation to the position of the hip, a relationship is found between the measurements obtained in the foot at both the barometric level and the positioning. Future research directions will investigate this finding.

The different study designs make it difficult to compare the results. We have been able to verify that there exists a relationship between the foot-, genus-, and pelvis-related parameters. However, the relationship with the stomatognathic system has been weaker but quite interesting for the interpretation of the correlation of the posture and the dental malocclusion. In this study, we found that the contact surface and oscillations of the centre of gravity at the antero-posterior level were correlated in the plantigrade phase. Proofs of a relationship between dental malocclusions and sagittal oscillation were not found.

What is being measured in this study is not real balancing, but its effect as represented by the movement of the centre of pressure on the platform. We found that Angle’s Class II is related with an anteriorised centre of gravity, while in Class I and III the centre of gravity is posterior. In line with Nobili at al., the subjects with Class II malocclusion show alterations in their posture in an anterior plane, while those with Class III malocclusion show a posteriorly displaced posture [9]. On the contrary, Ferrario et al. analysed postural balancing in subjects with foot orthotics and altered occlusal positions and declared that there was not a correlation between the displacement of the centre of pressures and postural balancing [13]. However, Julia-Sanchez et al. showed that the mandibular position has a significant influence on the control of the balance. The body equilibrium was greater when dental occlusion was established with rolls of cotton in the mandibular condition in comparison with the intercuspal position [38]. Oiea et al. showed that occlusal contact is one of the factors that affects the fluctuation of gravity. The appropriate occlusion achieved, maintaining a uniform occlusal contact in the posterior region, is crucial for the fluctuation of gravity [39]. Scharnweber et al. noted that blocking the occlusion leads to a reduction in the oscillation [40]. Although we have found significant associations between the variables studied, more research is required to establish standard values for postural control and the distribution parameters of plantar pressure, and to be able to evaluate and interpret them.

We accept that there are limitations in this study. An important limitation of our analysis is the lack of radiographic studies at the level of the pelvis and the trunk’s skeletal structures. These would provide information impossible to obtain by other means. In the future, we will increase the multidisciplinary research with physiotherapists, which will enable the valuing of other structural compensations, and hopefully, we will find more interesting results. It is necessary to follow up this cut at the longitudinal level to value the possible variations at the postural level and thus be able to determine which parameters are circumstantial and which are conclusive. Another limitation of the study could be the evaluation of children of different ages. Since occlusal stability changes from 8 to 14 years due to different dentitions and the same happens with plantar pressure and body posture, owing to the growth of the body, it is difficult to observe a strong relationship because of the change in individuals. Furthermore, since this is an observational study, only one correlation can be hypothesised. It is of interest to evaluate in subsequent research the relationship between dental malocclusion and body posture at different ages and how it changes.

## 5. Conclusions

The children showed a strong correlation between the structures that make up the biomechanics of the lower limb—hip, knee, and foot. On the other hand, a direct relationship is not found between the stomatognathic system and the structures of the lower limb (hip, knee, and foot). Having compared the dental classification with the baricentre, significant data were found related with the contact surface, especially in the plantigrade phase, and fundamentally with the centre of gravity. A predominance of the anteriority of the centre of gravity in Angle’s Class II is revealed.

## Figures and Tables

**Table 1:** Participants descriptive characteristics and anthropocentric data and foot measurements (*N* = 289).

Anthropocentric Data and Foot Measurements	Average (*N* = 289)	SD (*N* = 289)	95% CI	Correlation Coefficient
Age	10.83	1.43	10.66–11.00	
BMI Kg/m^2^	18.04	3.08	17.68–18.40	
Right FPI	5.04	2.27	4.47–5.30	0.000
Left FPI	4.83	2.29	4.56–5.09	r = 0.883
Right scaphoid height	2.57	0.76	2.48–2.66	0.000
Left scaphoid height	2.58	0.72	2.49–2.66	r = 0.875
Right global strength	51.08	6.51	50.32–51.84	0.000
Left global strength	48.91	6.52	48.15–49.67	r = −0.999
Pressure 1° right metatarsal	136.09	124.17	121.62–150.57	0.000
Pressure 1° left metatarsal	226.45	133.12	210.93–241.98	r = 0.512
Pressure 5° right metatarsal	333.06	112.65	319.293–346.20	0.000
Pressure 5° left metatarsal	109.70	109.40	96.94–122.46	r = 0.340
Pressure right heel	471.45	155.95	453.26–489.63	0.000
Pressure left heel	528.64	143.94	511.86–545.43	r = 0.286
Support phase of right heel	95.65	50.38	88.81–100.48	0.000
Support phase of left heel	90.98	52.14	84.95–97.02	r = 0.415
Right plantigrade phase	264.71	102.08	252.90–276.53	0.000
Left plantigrade phase	259.21	88.02	249.01–269.40	r = 0.469
Right propulsive phase	341.54	83.60	331.86–351.22	0.000
Left propulsive phase	345.88	83.30	336.24–355.53	r = 0.519
Surface of right contact	61,74	16.41	59.87–63.61	0.000
Surface of left contact	57.40	17.07	55.44–59.38	r = 0.778

CI—confidence interval, FPI—Foot Posture Index, SD—standard deviation, r—correlation coefficient.

**Table 2:** Mouth valuation of the participants, angle class, and correlation between participants.

**Angle Class**	**Angle’s Class I** **(*N* = 289)**	**Angle’s Class II** **(*N* = 289)**	**Angle’s Class III** **(*N* = 289)**
Right molar Angle class	29.1% *n* = 84	60.89% *n* = 176	10% *n* = 29
Left molar Angle class	21.1% *n* = 61	70.6% n = 204	8.3% *n* = 24
Right canine Angle class	46.36% *n* = 134	44.63% *n* = 129	9% *n* = 26
Left canine Angle class	40.1% *n* = 116	50.5% *n* = 146	9.3% *n* = 27
	**X^2^**	**Cramer’s V**	
Right molar–Left molar	0.000	0.807	
Right molar–Right canine	0.000	0.552	
Left Molar–Left canine	0.000	0.695	
Right canine–Left canine	0.000	0.847	

CI—confidence interval, number (percentage), SD—standard deviation, X^2^—Pearson chi-squared, Cramer’s V test.

**Table 3:** Correlation and inferential analysis between the Angle class and the foot’s biomechanics.

Foot’s Biomechanics	Right Molar *p*-Value	Left Molar *p*-Value	Right Canine *p*-Value	Left Canine *p*-Value
Right FPI	0.209	0.061	0.115	0.244
Left FPI	0.159	0.036 *	0.318	0.502
Right scaphoid height	0.565	0.643	0.048 *	0.148
Left scaphoid height	0.585	0.278	0.981	0.739
Right global strength	0.780	0.871	0.110	0.079
Left global strength	0.780	0.871	0.110	0.079
Pressure 1° right metatarsal	0.053	0.021	0.229	0.450
Pressure 1° left metatarsal	0.937	0.541	0.850	0.411
Pressure 5° right metatarsal	0.096	0.001	0.718	0.219
Pressure 5° left metatarsal	0.180	0.270	0.550	0.720
Right heel pressure	0.013	0.558	0.045 *	0.045 *
Left heel pressure	0.270	0.098	0.100	0.673
Right heel support phase	0.264	0.334	0.334	0.530
Left heel support phase	0.647	0.824	0.008 **	0.133
Right plantigrade phase	0.320	0.007 **	0.009 **	0.024 *
Left plantigrade phase	0.256	0.021 **	0.050 *	0.029 *
Right propulsive phase	0.402	0.699	0.323	0.735
Left propulsive phase	0.001 **	0.005 **	0.003 **	0.173
Right contact surface	0.014 *	0.044 *	0.191	0.284
Left contact surface	0.001 **	0.011 *	0.030 *	0.007 **
Centre of gravity	0.0001 ***	0.0001 ***	0.0001 ***	0.0001 ***

FPI—Foot Posture Index, * Kruskal–Wallis Test. Significance set at *p* < 0.05. * *p* < 0.05; ** *p* < 0.01; *** *p* < 0.001.

**Table 4:** Correlation between molar and canine class and centre of gravity of participants (*N* = 289).

Angle Class	Front *N* (%)	Back *N* (%)	Central *N* (%)	Strength Association £
Right Molar				
Class I	34 (17.1)	49 (58.3)	3 (50)	*p* = 0.0001 ***
Class II	162 (81.4)	10 (11.9)	3 (50)	0.439
Class III	3 (1.5)	25 (29.8)	0	
Left Molar				
Class I	16 (8.0)	44 (52.4)	1 (16.7)	*p* = 0.0001 ***
Class II	182 (91.5)	17 (20.2)	5 (83.3)	0.519
Class III	1 (0.5)	23 (27.4)	0	
Right Canine				
Class I	72(36.2)	56 (66.7)	5 (83.3)	*p* = 0.0001 ***
Class II	122 (61.3)	6 (7.1)	1 (16.7)	0.355
Class III	5(2.5)	22 (26.2)	0	
Left Canine				
Class I	62 (32.2)	51 (60.7)	3 (50)	*p* = 0.0001 ***
Class II	136 (68.3)	7 (8.3)	3 (50)	0.470
Class III	1 (0.5)	26 (31.0)	0(0)	

Number (percentage), £ Cramer’s V test. *** *p* < 0.001
